# Hyperuricemia and its related histopathological features on renal biopsy

**DOI:** 10.1186/s12882-019-1275-4

**Published:** 2019-03-18

**Authors:** Shulei Fan, Ping Zhang, Amanda Ying Wang, Xia Wang, Li Wang, Guisen Li, Daqing Hong

**Affiliations:** 1 Renal Department and Nephrology Institute, Sichuan Provincial People’s Hospital, School of Medicine, University of Electronic Science and Technology of China, Chengdu, 610072 China; 2 The George Institute for Global Health, University of NSW, Missenden Road, PO Box M201, Sydney, NSW 2050 Australia; 3 Department of Renal Medicine, Northern Beaches Hospital, Sydney, NSW 2086 Australia; 4 North Sichuan Medical College, Nanchong, 637000 China

**Keywords:** Hyperuricemia, Histopathological features, Chronic kidney disease

## Abstract

**Background:**

Hyperuricemia (HUA) is very common in chronic kidney disease (CKD). HUA is associated with an increased risk of cardiovascular events and accelerates the progression of CKD. Our study aimed to explore the relationship between baseline serum uric acid levels and renal histopathological features.

**Methods:**

One thousand seventy patients receiving renal biopsy in our center were involved in our study. The baseline characteristics at the time of the kidney biopsy were collected from Renal Treatment System (RTS) database, including age, gender, serum uric acid (UA), glomerular filtration rate (eGFR), serum creatinine (Cr), urea, albumin (Alb), 24 h urine protein quantitation (24 h-u-pro) and blood pressure (BP). Pathological morphological changes were evaluated by two pathologists independently. Statistical analysis was done using SPSS 21.0.

**Results:**

Among 1070 patients, 429 had IgA nephropathy (IgAN), 641 had non-IgAN. The incidence of HUA was 38.8% (*n* = 415), 43.8% (*n* = 188), and 43.2% (*n* = 277) in all patients, patients with IgAN and non-IgAN patients, respectively. Serum uric acid was correlated with eGFR (*r* = − 0.418, *p* < 0.001), Cr (*r* = 0.391, *p* < 0.001), urea (*r* = 0.410, *p* < 0.001), 24-u-pro (*r* = 0.077, *p* = 0.022), systolic blood pressure (SBP) (*r* = 0.175, *p* < 0.001) and diastolic blood pressure (DBP) (*r* = 0.109, *p* = 0.001). Multivariate logistic regression analysis showed that after adjustment for Cr, age and blood pressure, HUA was a risk factor for segmental glomerulosclerosis (OR = 1.800, 95% CI:1.309–2.477) and tubular atrophy/interstitial fibrosis (OR = 1.802, 95% CI:1.005–3.232). HUA increased the area under curve (AUC) in diagnosis of segmental glomerulosclerosis.

**Conclusions:**

Hyperuricemia is prevalent in CKD. The serum uric acid level correlates not only with clinical renal injury indexes, but also with renal pathology. Hyperuricemia is an independent risk factor for segmental glomerulosclerosis and tubular atrophy/interstitial fibrosis.

## Background

Uric acid is the product of purine metabolism in human body. 70% of uric acid in human body is excreted through kidneys. Uric acid is an intracellular oxidant when it is beyond the physiological range [1]. Hyperuricemia (HUA) is associated with endothelial dysfunction, vascular smooth muscle proliferation and interstitial inflammatory infiltration through a variety of mechanisms, such as inducing intracellular oxidative stress, mitochondrial dysfunction, inflammatory response and activation of the renin-angiotensin system (RAS) [2–7].

Hyperuricemia is a common phenomenon in patients with chronic kidney disease (CKD). Previous studies have shown that hyperuricemia was a risk factor for CKD [8, 9]. It can accelerate the progression of CKD [10–13], and increase the incidence of cardiovascular, cerebrovascular diseases and metabolic diseases [14–17]. However, the correlation between hyperuricemia and renal pathological changes is not entirely clear.

Previous studies suggested that HUA was associated with tubular interstitial lesions, and high uric acid levels indicated tubular interstitial lesions [18, 19]. However, the correlation between uric acid levels and glomerular sclerosis has not been studied. Our study aimed to investigate the correlation between uric acid and renal pathological changes, including both glomerular sclerosis and tubular interstitial lesions.

## Methods

### Study participants and data collection

Participants receiving renal biopsy in Sichuan Provincial People’s Hospital from January 2010 to December 2016 were screened. Those with adequate information on baseline characteristics in our Renal Treatment System (RTS) database were included in the current study. Exclusion criteria included inability to provide consent, enrollment in competing studies, pregnancy, familial hyperuricemia, transient hyperuricemia, primary gout, transient tubular injury, malignant hypertension, renal cancer, cirrhosis, recent chemotherapy or immunosuppressive therapy, organ transplantation, or dialysis treatment. A total of 1070 individuals (516 males and 554 females) were included in this study. The baseline demographic and clinical characteristics were collected at the time of renal biopsy from RTS database, including age, gender, serum uric acid, glomerular filtration rate (eGFR), serum creatinine, urea, albumin and 24 h urine protein quantitation (24 h-u-pro) and blood pressure. eGFR was estimated with CKD-EPI (CKD Epidemiology Collaboration) creatinine equation [20].

The study was approved by the Ethics Committee of the Sichuan Provincial People’s Hospital (Chengdu, China, No.2017–124). The de-identified data was obtained from RTS database. All patients gave fully informed written consent.

### Diagnosis criteria

Hyperuricemia was defined as a fasting serum uric acid level greater than 420 μmol/L (7 mg/dl) for male and greater than 357 μmol/L (6 mg/dl) for female participants [21].

Renal pathological diagnosis was reviewed independently by two renal pathologists who were blinded to previous pathology reports and patients’ clinical outcomes. Segmental sclerosis of glomerulus was classified as segmental glomerulosclerosis group (S0) and non- segmental glomerulosclerosis group (S1). On the basis of extent of tubular atrophy/interstitial fibrosis, patients were divided into mild injury (T1), moderate injury (T2), and severe injury (T3) according to current literatures (0–25%, 26–50, > 50%) [22, 23].

### Statistical analysis

Continuous data were presented as mean with standard deviation (SD) or median with interquartile ranges (IQR). Categorical variables were presented as proportions. Continuous data were compared by t-test or one-way ANOVA. Chi-square test was used to compare categorical variables between two groups. Pearson or Spearson correlation analysis was performed to calculate the correlation between uric acid and other clinical indicators. Logistic regression analysis was used to examine whether HUA was an independent predictor of segmental glomerulosclerosis or tubular atrophy/interstitial fibrosis. We also did sensitivity analyses to assess relationship between HUA and segmental glomerulosclerosis or tubular atrophy/interstitial fibrosis in several models. Receiver Operating characteristic Curves (ROC) was used and the area under curve (AUC) was analyzed to test whether HUA can increase the ability to diagnose glomerular segmental sclerosis and tubular atrophy/interstitial fibrosis. All analyses were performed using SPSS, version21.0. *p* value of less than 0.05 was considered statistically significant.

## Results

### Baseline clinical characteristics and pathological features

**Fig. 1:**
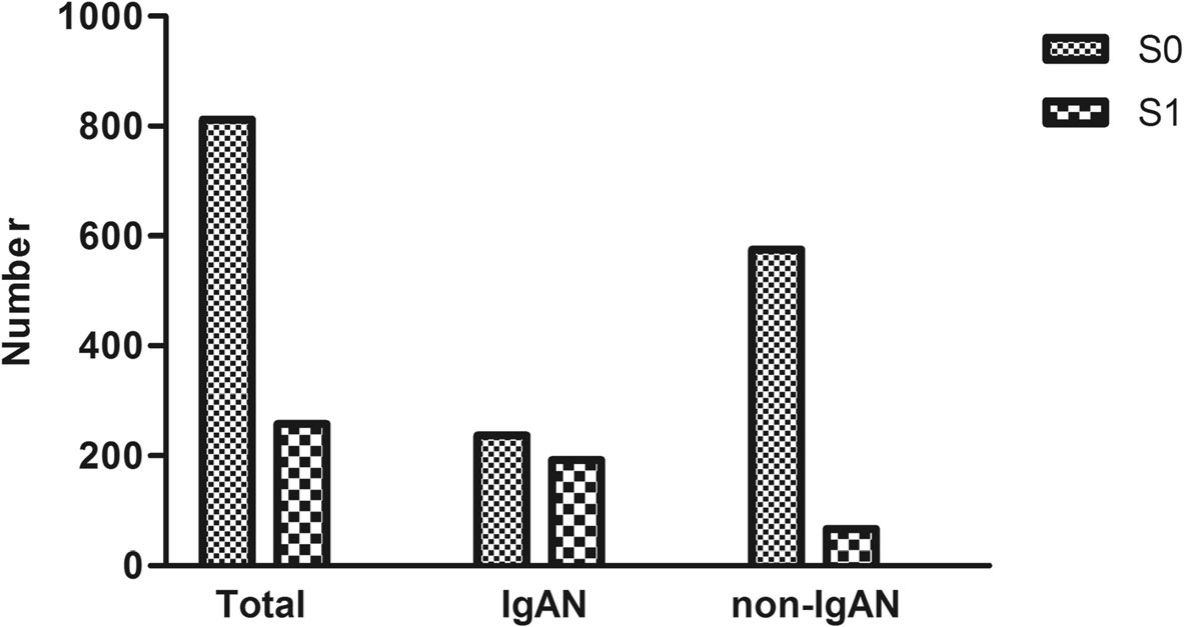
Distribution of segmental glomerular sclerosis. Notes: Number: number of patients. S0: non-segmental glomerular sclerosis. S1: segmental glomerular sclerosis

**Fig. 2:**
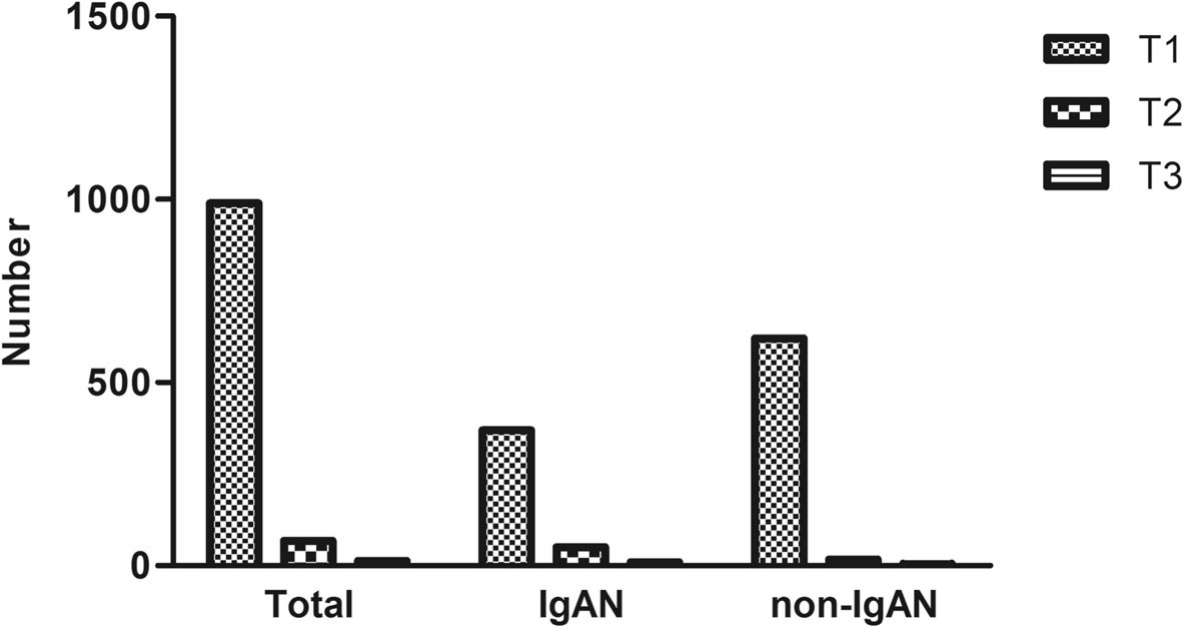
Distribution tubular atrophy / interstitial fibrosis. Notes: Number: number of patients. T1: mild tubular atrophy / interstitial fibrosis. T2: moderate tubular atrophy / interstitial fibrosis. T3: severe tubular atrophy / interstitial fibrosis

In the whole cohort, 429 (171 males and 258 females) of 1070 (516 males and 554 females) patients had biopsy proven IgAN. Patients with IgAN were younger, female predominant, had worse renal function, higher serum albumin level and lower 24 h-u-pro level, as compared to those with non-IgAN. The prevalence of hyperuricemia was 38.8% (*n* = 415), 43.8% (*n* = 188), and 43.2% (*n* = 277) in all patients, patients with IgAN and non-IgAN patients, respectively (*p* = 0.84, Table 1). Among the all participants (*n* = 1070), the majority of patients (812(75.9%)) did not have segmental glomerulosclerosis (Table 1, Fig. 1). The prevalence of tubular atrophy/interstitial fibrosis was 989 (92.4%), 68 (6.4%) and 13 (1.2%) for mild tubular atrophy/interstitial fibrosis, moderate tubular atrophy/interstitial fibrosis and severe tubular atrophy/interstitial fibrosis, respectively (Table 1, Fig. 2). The patients with IgAN had a higher ratio of segmental glomerulosclerosis and more serious situation of tubular atrophy/interstitial fibrosis than non-IgAN group (*P* < 0.001, Table 1).

**Table 1:** Baseline clinical characteristics and pathological features

	Total	IgAN	non-IgAN	*p*-value
	*n* = 1070	*n* = 429	*n* = 641	
Age (years)	38 ± 15	34 ± 12	40 ± 16	<0.001
Male (n, %)	516 (48.2%)	171 (39.9%)	345 (53.8%)	<0.001
Cr (μmol/L)	84.2 ± 50.1	90.3 ± 50.8	80.1 ± 50.4	0.004
eGFR (ml/min/1.73m^2^)	98.1 ± 31.3	93.0 ± 32.9	101.6 ± 29.8	<0.001
Urea (mmol/L)	6.5 ± 3.7	6.8 ± 3.7	6.3 ± 3.7	0.03
Alb (g/L)	33.2 ± 9.5	38.0 ± 6.7	30.0 ± 9.7	<0.001
UA (μmol/L)	372.7 ± 104.1	382.4 ± 105.2	366.2 ± 102.8	0.01
HUA (n,%)	415 (38.8%)	188 (43.8%)	277 (35.4%)	0.84
24 h-u-pro (g/d)	1.6 (0.5,4.0)	1.2 (0.5,2.3)	2.2 (0.5,5.1)	<0.001
SBP	126.74 ± 17.87	126.13 ± 17.52	127.13 ± 18.09	0.40
DBP	78.01 ± 12.32	77.72 ± 17.16	78.19 ± 12.43	0.57
hypertension(n,%)	230 (21.5%)	85 (19.8%)	145 (22.6%)	0.60
Histopathological changes				
S0 (n,%)	812 (75.9%)	237 (55.2%)	575 (89.7%)	<0.001
S1 (n,%)	258 (24.1%)	192 (44.8%)	66 (10.3%)	
T1 (n,%)	989 (92.4%)	369 (86.0%)	620 (96.7%)	<0.001
T2 (n,%)	68 (6.4%)	51 (11.9%)	17 (2.7%)	
T3 (n,%)	13 (1.2%)	9 (2.1%)	4 (0.6%)	

Notes: *Cr* creatinine, *eGFR* estimated glomerular filtration rate, *Alb* albumin, *UA* uric acid, *HUA* hyperuricemia, *24 h-u-pro* 24 h protein quantitation, *SBP* systolic blood pressure, *DBP* diastolic blood pressure, S0: non-segmental glomerular sclerosis, S1: segmental glomerular sclerosis, T1: mild tubular atrophy/interstitial fibrosis, T2: moderate tubular atrophy/interstitial fibrosis, T3: severe tubular atrophy/interstitial fibrosis

### Uric acid and renal pathological features

Participants with segmental glomerulosclerosis had higher level of uric acid and worse renal function than those without segmental glomerulosclerosis in the whole cohort, IgAN and non-IgAN cohort (Table 2). In the whole cohort, the number of mild tubular atrophy/interstitial fibrosis, moderate tubular atrophy/interstitial fibrosis and severe tubular atrophy/interstitial fibrosis was 989, 68 and 13, respectively. Patients whose tubular interstitial lesions were more serious, had higher uric acid and lower renal function (Table 2). Among the 1070 patients, uric acid was correlated with eGFR (*r* = − 0.418, *p* < 0.001), Cr (*r* = 0.391, *p* < 0.001), urea (*r* = 0.410, *p* < 0.001), 24-u-pro (*r* = 0.077, *p* = 0.022), systolic blood pressure (*r* = 0.175, *p* < 0.001) and diastolic blood pressure (*r* = 0.109, *p* = 0.001). Uric acid was also correlated with segmental glomerulosclerosis (*r* = 0.117, *P* < 0.001) and tubular atrophy/interstitial fibrosis (*r* = 0.190, *P* < 0.001) in the whole cohort.

**Table 2:** Uric acid and renal pathological features

Total	S0	S1	*p*-value	T1	T2	T3	*p*-value
(*n* = 1070)	(*n* = 812)	(*n* = 258)		(*n* = 989)	(*n* = 68)	(*n* = 13)	
age	38.67 ± 15.51	34.87 ± 11.53	<0.001	37.64 ± 14.99	38.91 ± 11.54	41.15 ± 9.54	0.55
SBP	126.42 ± 17.55	127.74 ± 18.84	0.33	126.24 ± 17.79	131.18 ± 15.83△	142.17 ± 24.07#	0.001
DBP	77.62 ± 11.98	79.23 ± 13.29	0.09	77.67 ± 12.29	81.05 ± 10.00△	88.08 ± 18.46	0.002
UA	365.8 ± 102.2	394.3 ± 107.1	<0.001	367.0 ± 101.3	436.6 ± 114.1△	469.1 ± 103.0#	<0.001
Cr	81.5 ± 52.7	92.7 ± 43.2	0.002	78.1 ± 40.7	146.2 ± 78.0△	221.8 ± 117.4#	<0.001
eGFR	101.0 ± 30.4	89.2 ± 32.7	<0.001	102.0 ± 28.7	54.5 ± 23.5△	35.3 ± 18.1#ο	<0.001
Urea	6.3 ± 3.7	7.2 ± 3.6	<0.001	6.2 ± 3.3	9.9 ± 5.6△	14.0 ± 5.5#	<0.001
Alb	32.0 ± 9.8	37.1 ± 7.3	<0.001	33.0 ± 9.7	35.2 ± 7.4	35.6 ± 6.1	0.12
24 h-u-pro	1.7 (0.4, 4.3)	1.5 (0.7, 2.8)	<0.001	1.6 (0.5, 4.0)	1.9 (1.0, 3.6)	3.7 (1.9, 4.6)	0.89
IgAN(*n* = 429)	(*n* = 237)	(*n* = 192)		(*n* = 369)	(*n* = 51)	(*n* = 9)	
age	35.23 ± 12.30	33.22 ± 10.25	0.07	34.09 ± 11.77	35.73 ± 9.73	36.78 ± 6.03	0.51
SBP	126.73 ± 16.64	125.43 ± 18.53	0.48	125.21 ± 17.48	130.74 ± 15.12	136.88 ± 24.86	0.03
DBP	77.35 ± 11.30	78.16 ± 13.13	0.53	76.95 ± 12.06	81.53 ± 10.09△	87.00 ± 19.03	0.006
UA	374.6 ± 101.2	392.0 ± 109.5	0.09	373.1 ± 101.1	435.3 ± 115.3△	464.9 ± 97.2#	<0.001
Cr	89.7 ± 56.6	91.0 ± 42.8	0.79	79.6 ± 33.0	152.4 ± 86.2△	177.6 ± 57.4#	<0.001
eGFR	94.9 ± 33.0	90.7 ± 32.6	0.19	99.6 ± 29.2	53.9 ± 24.4△	42.2 ± 16.4#	<0.001
Urea	6.6 ± 3.7	7.1 ± 3.7	0.11	6.2 ± 2.8	10.1 ± 6.0△	13.6 ± 4.5#	<0.001
Alb	37.7 ± 7.5	38.6 ± 5.6	0.17	38.4 ± 6.7	36.1 ± 6.9△	34.9 ± 4.5	0.02
24 h-u-pro	1.0 (0.4, 2.0)	1.4 (0.7, 2.5)	0.52	1.0 (0.5, 2.1)	2.0 (0.9, 4.0)△	3.7 (2.0, 4.8)	<0.001
non-IgAN (*n* = 641)	(*n* = 575)	(*n* = 66)		(*n* = 620)	(*n* = 17)	(*n* = 4)	
age	40.09 ± 16.45	39.73 ± 13.60	0.84	39.75 ± 16.25	48.47 ± 11.49△	51.00 ± 8.98	0.03
SBP	126.31 ± 17.90	133.89 ± 18.40	0.002	126.81 ± 17.94	132.50 ± 18.40	152.75 ± 21.42#ο	0.009
DBP	77.72 ± 12.24	82.08 ± 13.38	0.009	78.07 ± 12.41	79.57 ± 9.95	90.25 ± 19.87	0.13
UA	362.2 ± 102.4	401. ± 100.3	0.003	363.4 ± 101.4	440.4 ± 113.6△	478.6 ± 130.7#	<0.001
Cr	78.1 ± 50.6	97.7 ± 44.5	0.003	77.3 ± 44.7	127.3 ± 41.5△	321.3 ± 165.3	<0.001
eGFR	103.5 ± 28.9	84.7 ± 32.6	<0.001	103.4 ± 28.3	56.4 ± 20.9△	19.8 ± 11.3#ο	<0.001
Urea	6.2 ± 3.7	7.3 ± 3.4	0.02	6.2 ± 3.6	9.5 ± 3.9△	14.7 ± 8.0	<0.001
Alb	29.6 ± 9.7	32.6 ± 9.5	0.02	29.8 ± 9.7	32.6 ± 8.4	37.1 ± 9.6	0.17
24 h-u-pro	2.3 (0.4, 5.1)	1.8 (1.0, 4.5)	0.44	2.3 (0.4, 5.1)	1.6 (1.1, 4.8)	3.3 (1.8, 3.3)	0.86

Notes: △:T1 vs.T2, *p*<0.05, #: T1 vs. T3, *p*<0.05, Ο: T2 vs. T3, *p*<0.05, *UA* uric acid, *Cr* creatinine, *eGFR* estimated glomerular filtration rate, *Alb* albumin, *24 h-u-pro* 24 h protein quantitation, *SBP* systolic blood pressure, *DBP* diastolic blood pressure

### Hyperuricemia and renal pathological changes

Univariate logistic regression analysis showed that hyperuricemia was associated with segmental glomerulosclerosis (OR = 1.918, 95% CI:1.444–2.546) and tubular atrophy/interstitial fibrosis (OR = 3.279, 95% CI:2.037–5.276). Multivariate logistic regression analysis confirmed this finding (segmental glomerulosclerosis (OR = 1.800, 95% CI:1.309–2.477) (Table 3) and tubular atrophy/interstitial fibrosis (OR = 1.802, 95% CI:1.005–3.232)) after adjustment for serum creatinine, age and blood pressure. Furthermore, hyperuricemia remained a risk factor for segmental glomerulosclerosis after adjustment for other models, such as Cr + 24 h-u-pro + age + BP, Cr + Alb + age + BP, eGFR +Alb + age + BP (Table 3).

**Table 3:** Logistic analysis for predictors of segmental glomerulosclerosis

	OR^1^ (95% CI)	OR^2^ (95% CI)	OR^3^ (95% CI)	OR^4^ (95% CI)	OR ^5^ (95% CI)
HUA	1.800△ (1.309–2.477)	1.771△ (1.250–2.509)	1.812△ (1.297–2.533)	1.400 (0.975–2.011)	1.422△ (1.003–2.016)

Notes: △: *p*<0.05, OR^1^: adjusted for Cr + age + BP, OR^2^: adjusted for Cr + 24 h-u-pro + age + BP, OR^3^: adjusted for Cr + Alb + age + BP, OR^4^: adjusted for eGFR + 24 h-u-pro + age + BP, OR^5^: adjusted for eGFR +Alb + age + BP, *HUA* hyperuricemia, *Cr* creatinine, *eGFR* estimated glomerular filtration rate, *Alb* albumin, *24 h-u-pro* 24 h protein quantitation, *BP* blood pressure

**Fig. 3:**
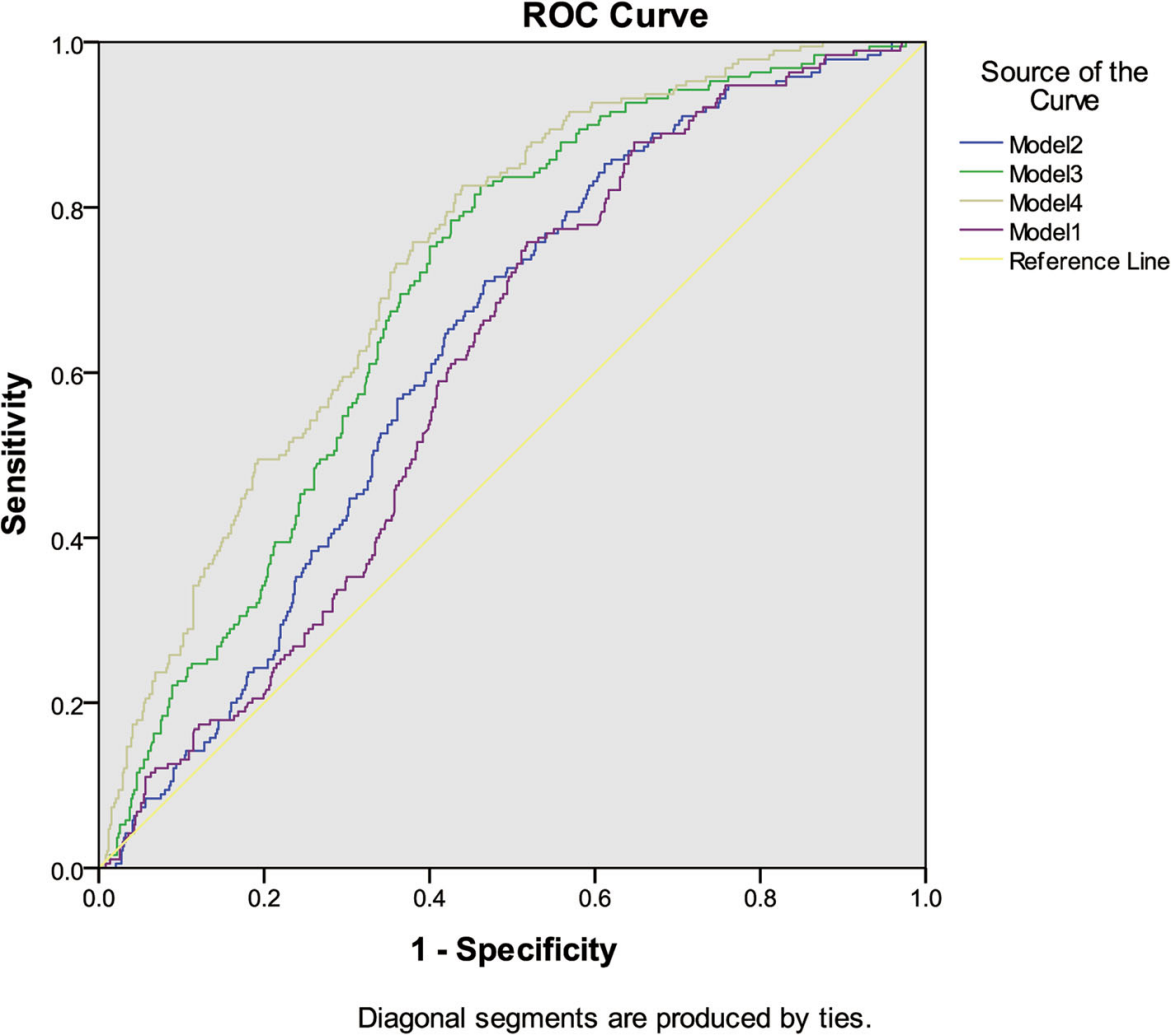
Diagnosis of segmental glomerular sclerosis without HUA. Notes: Model 1: Cr + age + BP. Model 2: Cr + 24 h-u-pro + age + BP. Model 3: Cr + Alb + age + BP. Model 4: eGFR +Alb + age + BP

**Fig. 4:**
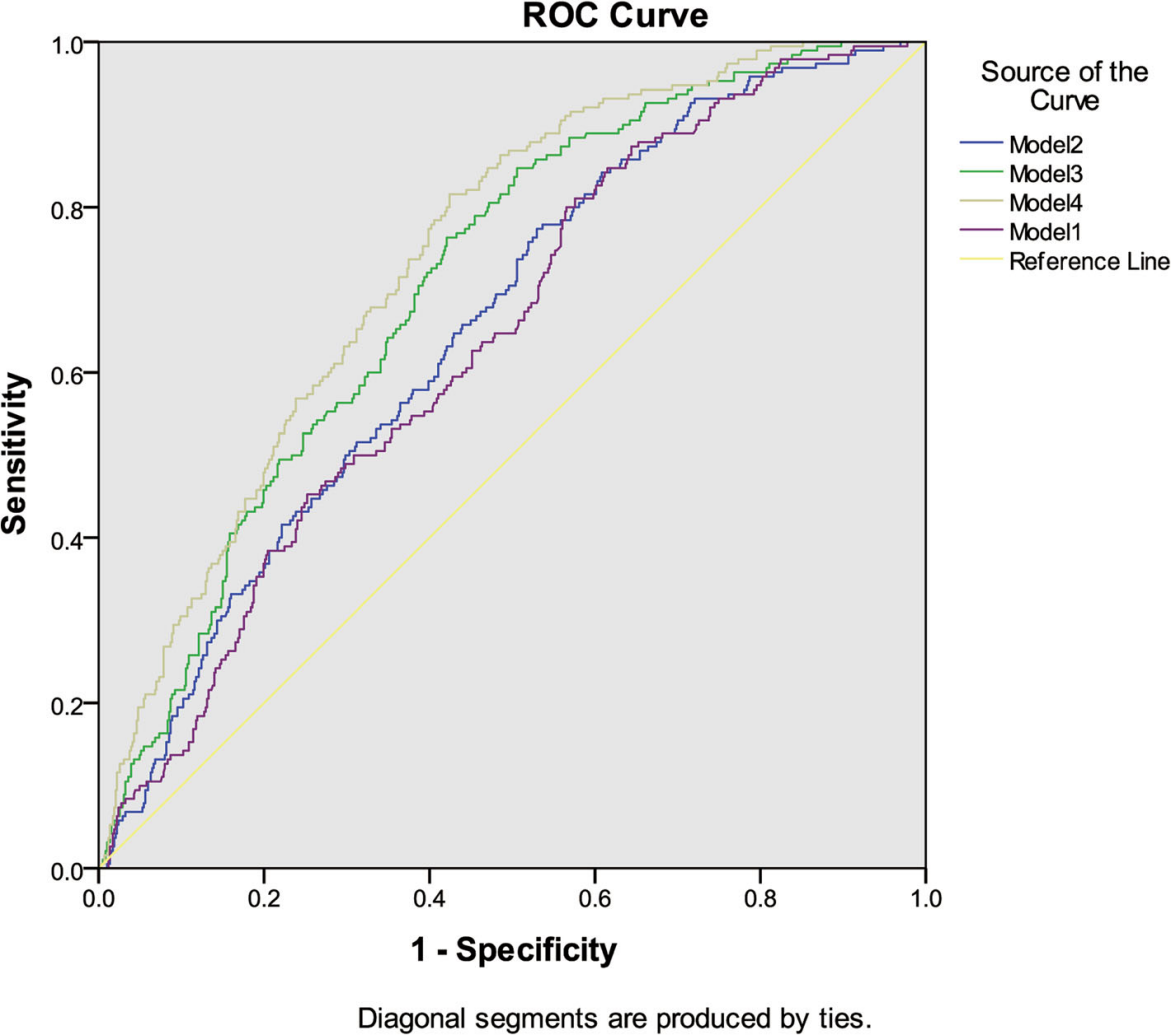
Diagnosis of segmental glomerular sclerosis with HUA. Notes: Model 1: HUA + Cr + age + BP. Model 2: HUA + Cr + 24 h-u-pro + age + BP. Model 3: HUA + Cr + Alb + age + BP. Model 4: HUA + eGFR +Alb + age + BP

The predictors, which were statistical significant from logistic regression analysis, were used to study their ability to diagnose segmental glomerulosclerosis with Receiver Operating Characteristic curves in four models. We analyzed the area under the curve and compared the difference between models with HUA and models without HUA. When considering the variable of hyperuricemia, the area under the curve was larger than that without hyperuricemia (Figs. 3 & 4, Table 4). Compared with the other three models, model 4 (HUA + eGFR + Alb + age + BP) had the largest area under the curve. In model 4, AUC changed from 0.738 to 0.741 after adding hyperuricemia to the model (Table 4).

**Table 4:** Specificity and sensitivity for predicting segmental glomerulosclerosis

	Model 1		Model 2		Model 3		Model 4	
	HUA	HUA	HUA	HUA	HUA	HUA	HUA	HUA
	absent	present	absent	present	absent	present	absent	present
Sensitivity	0.758	0.815	0.711	0.773	0.837	0.774	0.828	0.819
Specificity	0.493	0.429	0.531	0.466	0.535	0.571	0.559	0.573
AUC (SE)	0.617 (0.020)	0.6433 (0.020)	0.629 (0.021)	0.652 (0.021)	0.700 (0.019)	0.716 (0.019)	0.738 (0.019)	0.741 (0.019)

Notes: *HUA* hyperuricemia, *Cr* creatinine, *eGFR* estimated glomerular filtration rate, *Alb* albumin, *24 h-u-pro* 24 h protein quantitation, *BP* blood pressure. Model 1: Cr + age + BP, Model 2: Cr + 24 h-u-pro + age + BP, Model 3: Cr + Alb + age + BP, Model 4: eGFR +Alb + age + BP, *AUC* area under the curve, *SE* standard error

## Discussion

Our study included 1070 patients with chronic kidney disease who received renal biopsy. The overall prevalence of HUA was 38.8%, suggesting that uric acid lowering treatment may be beneficial for more than one third of the patients. We attempted to divide the 1070 patients into different subgroups according to renal pathology, such as IgAN, membranous nephropathy (MN) group, focal segmental glomerulosclerosis (FSGS), etc. However, preliminary data analysis revealed that other groups except IgAN had similar clinical features in the current cohort. Moreover, the small number of cases of individual group, is not conducive to the statistical analysis. Finally, we divided all patients into IgAN and non-IgAN and found that the prevalence of HUA was higher in IgAN than in non-IgAN.

In the studied cohort, we found that the more serious the histological injury was, the worse renal function were, which were in accordance with previous studies [22–24]. We also found that uric acid was associated with renal pathological changes. High uric acid levels are associated with poorer kidney function. In order to further investigate the correlation between uric acid and histological damage of kidney, we performed logistic regression analysis for all patients. The results showed that after adjustment for Cr, age and blood pressure, HUA was still a risk factor for segmental glomerulosclerosis (OR = 1.800, 95% CI:1.309–2.477) and tubular atrophy/interstitial fibrosis (OR = 1.802,95% CI:1.005–3.232). Furthermore, we built four different models as sensitivity analysis, and found that HUA was still a risk factor for segmental glomerulosclerosis in all four models (Table 3). However, we did not find a significant association between HUA and tubular atrophy/interstitial fibrosis. In model 4, if the index of HUA was added, the area under curve increased from 0.738 to 0.741 (Table 4). Although this increase was not significant, it could improve the value of diagnosis to some extents.

In recent years, with the lifestyle modifications, the prevalence of hyperuricemia (HUA) is increasing, and the prevalence of HUA in Chinese adults ranged from 8.4 to 13.3% [25, 26]. Our study showed that patients with glomerulonephritis have an even higher prevalence of HUA, indicating a considerable number of population might benefit from uric acid lowering interventions. HUA is not only an independent risk factor for CKD [8, 9], but isalso associated with an increased risk of CKD progression [10, 11] and cardiovascular outcomes [14, 15]. Moreover, the renal pathological changes are also one of major prognostic predictors for CKD progression. The more serious the lesion is, the worse the renal prognosis is [22–24]. The pathological examination is deemed to be a gold standard for the evaluation of the extent of chronic kidney damages. However, it relies on renal biopsy, which is an invasive examination. In some clinical settings, this invasive method might be contraindicated in or refused by the patients. Looking for a clinical biochemical indicator to assist with evaluating the necessity of performing renal biopsy in guiding clinical management. Uric acid seems to be a potential indicator in this regard. After multiple logistic regression and sensitivity analyses, HUA was found independently associated with segmental glomerulosclerosis and tubular atrophy/interstitial fibrosis. This can be further validated in prospective studies in the future.

The relationship between HUA and renal pathological features could be explained by the mechanisms how HUA injures the kidneys. HUA can lead to injury in target organs, such as glomerular sclerosis, glomerular hypertension, glomerulosclerosis, interstitial lesions, and acute kidney injury [27]. HUA can also directly affect the renal interstitium and lead to fibrosis by inducing the transdifferentiation of glomerular epithelial cells [6]. Uric acid is closely related to the progression of kidney disease [28]. After the treatment of HUA, eGFR increased, proteinuria decreased and renal function improved [29–32]. Histological changes in kidney are associated with a variety of factors, not just uric acid [6]. The underlying implication of HUA and renal pathological changes could somehow be explained by uric acid metabolism in the kidney. The glomerulus is a mass of capillary network. Uric acid crystals are deposited in renal tubules and renal interstitium, causing kidney diseases. HUA can induce oxidative stress and endothelial dysfunction, causing renal vasoconstriction, glomerular hypertension, renal blood flow reduction [5, 7, 12]. It also activates the RAS system, leading to glomerulosclerosis and interstitial fibrosis [33, 34].

Due to the nature of retrospective cross-sectional study, there are some limitations in our study. Firstly, we were unable to draw a causal relationship between uric acid and renal pathological changes. Secondly, some confounders were not collected and included in our analyses, which may have an impact on the results. However, in our study, we found that HUA was associated with glomerulosclerosis and tubulointerstitial injury, which could be helpful in predicting glomerulosclerosis and tubulointerstitial injury in clinical practice especially for patients not going to or not willing to have renal biopsy. The results also raise that HUA as a potential treatment target as recommended by current guidelines might be helpful with renal sclerosis, which needs large scale prospective studies to prove.

## Conclusions

Hyperuricemia is prevalent in CKD. Uric acid correlates not only with clinical renal injury indexes, but also with renal pathology. Hyperuricemia is independently associated with segmental glomerulosclerosis and tubular atrophy/interstitial fibrosis.

## Abbreviations

24 h-u-pro: 24 h protein quantitation
Alb: Albumin
AUC: Area under curve
CKD: Chronic kidney disease
Cr: Creatinine
DBP: Diastolic blood pressure
eGFR: Glomerular filtration rate
HUA: Hyperuricemia
IgAN: IgA nephropathy
ROC: Receiver Operating characteristic Curves
RTS: Renal Treatment System
SBP: Systolic blood pressure
UA: Uric acid
